# Effective Connectivity between Ventral Occipito-Temporal and Ventral Inferior Frontal Cortex during Lexico-Semantic Processing. A Dynamic Causal Modeling Study

**DOI:** 10.3389/fnhum.2017.00325

**Published:** 2017-06-22

**Authors:** Marcela Perrone-Bertolotti, Louise Kauffmann, Cédric Pichat, Juan R. Vidal, Monica Baciu

**Affiliations:** ^1^Department of Psychology, Université Grenoble Alpes, CNRS, LPNC UMR 51055105Grenoble, France; ^2^Neural Mechanisms of Human Communication Research group, Max Planck Institute for Human Cognitive and Brain SciencesLeipzig, Germany

**Keywords:** dorsal stream, ventral stream, phonology, semantic, ventral occipito-temporal cortex, ventral inferior frontal cortex, dorsal inferior frontal cortex

## Abstract

It has been suggested that dorsal and ventral pathways support distinct aspects of language processing. Yet, the full extent of their involvement and their inter-regional connectivity in visual word recognition is still unknown. Studies suggest that they might reflect the dual-route model of reading, with the dorsal pathway more involved in grapho-phonological conversion during phonological tasks, and the ventral pathway performing lexico-semantic access during semantic tasks. Furthermore, this subdivision is also suggested at the level of the inferior frontal cortex, involving ventral and dorsal parts for lexico-semantic and phonological processing, respectively. In the present study, we assessed inter-regional brain connectivity and task-induced modulations of brain activity during a phoneme detection and semantic categorization tasks, using fMRI in healthy subject. We used a dynamic causal modeling approach to assess inter-regional connectivity and task demand modulation within the dorsal and ventral pathways, including the following network components: the ventral occipito-temporal cortex (vOTC; dorsal and ventral), the superior temporal gyrus (STG; dorsal), the dorsal inferior frontal gyrus (dIFG; dorsal), and the ventral IFG (vIFG; ventral). We report three distinct inter-regional interactions supporting orthographic information transfer from vOTC to other language regions (vOTC -> STG, vOTC -> vIFG and vOTC -> dIFG) regardless of task demands. Moreover, we found that (a) during semantic processing (direct ventral pathway) the vOTC -> vIFG connection strength specifically increased and (b) a lack of modulation of the vOTC -> dIFG connection strength by the task that could suggest a more general involvement of the dorsal pathway during visual word recognition. Results are discussed in terms of anatomo-functional connectivity of visual word recognition network.

## Introduction

Despite the significant amount of knowledge of the cerebral networks involved in visual word recognition (e.g., Price, 2012), yet little is known on the functional connections between the regions belonging these networks. One possible approach to evaluate these interactions in terms of effective connectivity and modulation by the language operations, is the Dynamic Causal Modeling (DCM; e.g., Mechelli et al., 2005; Price and Mechelli, 2005; Heim et al., 2009; David et al., 2011; Richardson et al., 2011; Yvert et al., 2012). In this study we were interested in better specifying the ventral and dorsal pathways involved in visual word recognition and assessing their modulation by lexico-semantic and phonological processes. We were also interested in the specific involvement of the ventral and dorsal inferior frontal gyrus (IFG) in relation of the lexico-semantic and phonological processing, respectively.

Phonologic and lexico-semantic operations are linguistic concepts encompassing variable sub-processes and supported by brain networks located predominantly in the left dominant hemisphere (Demonet et al., 2005; Vigneau et al., 2006, 2011; Price, 2012; Hervé et al., 2013). Phonological and lexico-semantic operations play a critical role in both spoken and written language and experimental data suggest that they are processed and relayed along two cerebral pathways. Studies of visual word recognition and reading suggest that phonological and lexico-semantic processing are implemented within distinct brain streams (Jobard et al., 2003; see Hickok and Poeppel, 2004; Saur et al., 2008 for speech processing). Indeed, a meta-analysis by Jobard et al. (2003) indicates that visual word recognition involves a dual-route process including a dorsal grapho-phonological stream or “surface” reading route, and a ventral lexico-semantic stream, or “deep” reading route (Jobard et al., 2003; Price, 2012; see also Richardson et al., 2011; Taylor et al., 2012).

During visual word processing, phonological, and semantic operations can be experimentally dissociated by manipulating item familiarity (e.g., single words or pseudo-words, Juphard et al., 2011) and task demands (e.g., phonological judgment, semantic categorization). For instance, if the task demand is phonological, processing will be focused on the grapho-phonemic conversion—GPC—(i.e., decoding visual input from letters to sounds or phonemes; Herbster et al., 1997; Fiez et al., 1999; Alario et al., 2003; Perrone-Bertolotti et al., 2011). GPC involves left ventral occipito-temporal cortex (vOTC, also called the “Visual Word Form Area”) which is highly word-like selective (Hamamé et al., 2013; Perrone-Bertolotti et al., 2014) and considered a central node in visual language processing (Dehaene et al., 2005, 2015; Vinckier et al., 2007; Cohen et al., 2008; see also Seghier et al., 2012). This region interacts with high-level language regions (such as the inferior frontal gyrus, Bitan et al., 2005; Vidal et al., 2012; Yvert et al., 2012; Perrone-Bertolotti et al., 2014; Schurz et al., 2014) and is modulated by top-down perceptual processes (Levy et al., 2013; Vidal et al., 2014). The GPC also involves the inferior parietal lobule (iPL) and the supramarginal gyrus (SMG; Jobard et al., 2003; Vigneau et al., 2006; Juphard et al., 2011; see also Stoeckel et al., 2009). Phoneme recognition and selection involves the superior temporal gyrus (STG; Pugh et al., 1996; Vandenberghe et al., 1996; Billingsley et al., 2001; Vigneau et al., 2006; Perrone-Bertolotti et al., 2011), while the activation of phoneme codes involves the inferior frontal gyrus (IFG; posterior/dorsal part, pars opercularis) and the supplementary motor area (SMA; Dapretto and Bookheimer, 1999; Poldrack et al., 1999; Burton, 2001; McDermott et al., 2003; Heim et al., 2005; Mainy et al., 2007; Tivarus et al., 2008; Turken and Dronkers, 2011). Anatomically, these temporo-parieto-frontal regions are connected by means of the superior longitudinal fasciculus; fronto-temporal regions are connected via the arcuate fasciculus (Saur et al., 2008; Friederici and Gierhan, 2013; Kellmeyer et al., 2013). Overall, the dorsal stream plays an important role in phonological and articulatory processes during visual words recognition (Richardson and Price, 2009; Menjot De Champfleur et al., 2013; Shinoura et al., 2013).

If the task demand is focused on the lexico-semantic processing, an activation of vOTC is also observed. Moreover, access and retrieval of lexical representations involve middle and inferior temporal areas (Billingsley et al., 2001; Gitelman et al., 2005), while the anterior temporal gyrus is important for semantic processing (Fujimaki et al., 2009; Binney et al., 2010; Visser et al., 2010; Yvert et al., 2012) and to convert modality-specific information into an amodal representation (Marinkovic et al., 2003; Lau et al., 2008). It has been suggested that the angular gyrus (Seghier et al., 2010; Price and Devlin, 2011) is involved in semantic memory (Price, 2000; Mechelli et al., 2007; Ben-Shalom and Poeppel, 2008). Finally studies pointed out the involvement of the anterior part of the IFG (anterior/ventral part at the junction between pars triangularis and pars orbitalis; (Mainy et al., 2008; Binder et al., 2009; Bedo et al., 2014) in semantic retrieval and higher level semantic process. Anatomical connections between these regions depend on the uncinate, inferior fronto-occipital and inferior longitudinal fasciculus (see Weiller et al., 2011). Overall, the ventral stream plays an important role in lexical retrieval and semantic processing (Yeatman et al., 2012; Cummine et al., 2013; Mahoney et al., 2013; Menjot De Champfleur et al., 2013; Shinoura et al., 2013).

Altogether, these studies suggest common involvement of vOTC and IFG for both lexico-semantic and phonological processing, with functional segregation of the parietal and temporal regions according to each type of information processing. Nevertheless, others studies suggest that the cerebral network involved in both lexico-semantic and phonological processes, is similar and only the intensity of brain activation (Juphard et al., 2011) and the connectivity between the key regions (Yvert et al., 2012) is different. In a previous study, we described the functional dynamics of phonological and lexico-semantic processes using DCM of event-related potentials (ERP) combined with source reconstruction, in order to evaluate the involvement of ventral and dorsal pathways (Yvert et al., 2012). Participants were instructed to perform phoneme detection in pseudo-words (phonological task) and semantic categorization of words (semantic task). Our ERP results showed common sequential activation of several predominantly left-hemisphere regions going from the occipito-temporal cortex to the superior, inferior and anterior temporal lobe (ATL), reaching the IFG. Moreover, DCM results indicated stronger connectivity within the ATL only during semantic task as a consequence of an increase in forward connectivity from the inferior temporal gyrus (ITG) to the ATL. Contrary to previously mentioned studies supporting a dual stream model for visual word processing, our results suggest the involvement of a common cerebral network with similar dynamics and connectivity for both phonological and lexico-semantic processes (see also Juphard et al., 2011). This however excludes the connectivity between ITG and ATL modulated by lexico-semantic processes (ventral semantic stream). Moreover, our results did not show phonological and lexico-semantic segregation within the IFG, as suggested by previous studies (e.g., Bokde et al., 2001; Mainy et al., 2007).

Indeed, the functional segregation within the IFG (opercularis vs. orbitalis) is still a matter of debate. Activity of ventral and dorsal IFG is selectively modulated by item type (word, pseudo-word) and the task demand (phonological or lexico-semantic; Demonet et al., 1992; Bookheimer, 2002; Bitan et al., 2005; Heim et al., 2005; Mainy et al., 2007). This specialization was also apparent in anatomical and functional connectivity with posterior regions (Bokde et al., 2001; Heim et al., 2005) such as vOTC (visual language input; e.g., Bouhali et al., 2014) and temporal cortex (e.g., Friederici and Gierhan, 2013). Using DCM, Bitan et al., 2005) suggested that the IFG plays a pivotal role in setting cognitive task demands, mediated through top-down modulation from the IFG to more task-selective regions (here, spelling and phonological tasks). They proposed that top-down influences from IFG could enhance the sensitivity of others specialized brain regions. However, this dissociation between dorsal and ventral IFG was not found by (Bitan et al., 2005) and other studies failed to show evidence supporting this possibility (e.g., Heim et al., 2009; Yvert et al., 2012).

In the current study we used fMRI study to evaluate the effective connectivity underlying word recognition for lexico-semantic (categorization task) and phonological (phoneme detection task) processes using a DCM approach. Our aim was to assess the activity modulation in ventral and dorsal pathways by lexico-semantic and phonological processes in a common cerebral network. We took advantage of the high spatial resolution of fMRI to identify two specific sub-regions in the inferior frontal gyrus, a dorsal region suggested as more involved in phonological processing and a ventral region suggested as more involved in semantic processing. Moreover, we evaluated the connectivity of these sub-regions with more posterior language regions. We expected to observe dorsal pathway connectivity modulation by phonological processing, including the dorsal inferior frontal gyrus (dIFG) and a ventral pathway connectivity modulation by lexico-semantic processing including the ventral inferior frontal gyrus (vIFG). According to previous studies, we expected to observe a significant connectivity modulation from the IFG sub-regions to the more posterior language regions reflecting active top-down modulation according to each of language information processing.

## Materials and methods

### Participants

Twenty-four participants (mean age = 26.91 years, SD = 3.43, max = 33 and min = 19 years; 12 females) took part in the experiment. They were all right-handed according to the Edinburgh Handedness Inventory (Oldfield, 1971) and French native speakers. They had normal or corrected-to-normal vision and no history of neurological, psychiatric disorders, learning disabilities or language disorders. All participants gave informed written consent for the experiment. The study was approved by the local Ethics Committee (CPP n°09-CHUG-14, 04/06/2009), which was in accordance with the Code of Ethics of the World Medical Association (Declaration of Helsinki) for experiments with humans.

### Stimuli

Two language tasks were used during two separate runs: a phonological (PHONO) task and a semantic (SEM) task. Each run included two conditions: “language” and “control.” The language condition of the PHONO run included 30 legal pseudo-words (PW), each one composed of six to seven letters. Half of the pseudo-words contained the phoneme /o/ corresponding to three orthographic forms in French, the graphemes /o/, /eau/ and /au/. The position of the three orthographical shapes (graphemes) of the phoneme /o/ was counterbalanced, in order to neutralize a possible bias related to orthographic visual representation (Cousin et al., 2009; Perrone-Bertolotti et al., 2011; Yvert et al., 2012). The language condition of the SEM run included 30 French words (W) of six to seven letters in length each. Words were of medium and high frequency and extracted from the *Lexique.org* database (New et al., 2004). Half of the words designated plants and animals (living items) and the other half designated objects (non-living items). The control condition was identical for the two runs, PHONO and SEM, and included 30 unreadable words (UW) written in unreadable characters (Karalyn Patterson font). All language stimuli were written in white Courier New font and control (UW) stimuli were written in white Karalyn Patterson font, size 40, and centered on black screen. They were generated by the E-Prime software (E-prime Psychology Software Tools Inc., Pittsburgh, USA), running on a PC computer. Each stimulus belonged to a trial (2.5 s duration) composed of 0.5 s fixation and 2 s stimulus (language or control).

### Tasks

**Figure 1:**
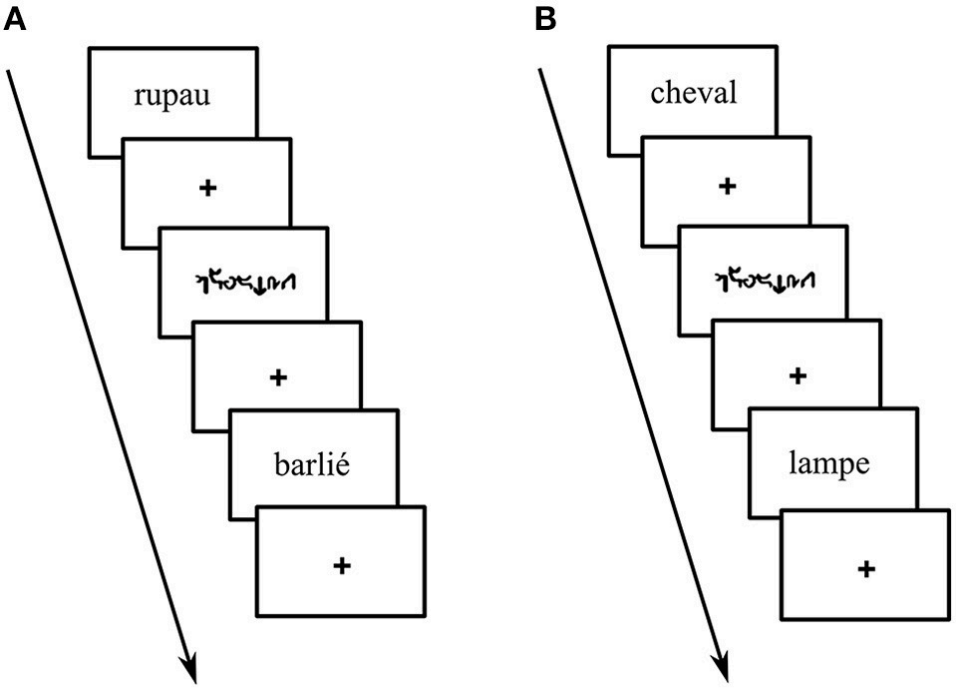
Example of stimuli presented during: **(A)** phoneme detection: pseudo-word with the target phoneme (e.g., “*rupau*,” here phoneme /o/ corresponding to grapheme “au”) or without the target phoneme (e.g., “*barlié*”) **(B)** living categorization: living words (e.g., “*cheval”* –horse-) or non-living words (e.g., “*lampe”* –lamp-) and unreadable control words (i.e., written in *Karalyn Patterson* font).

During the language condition of the PHONO run, participants had to perform a phoneme detection task (see Perrone-Bertolotti et al., 2011; Yvert et al., 2012). Specifically, they were required to judge whether or not the pseudo-words (PW) contained the sound /o/. During the language condition of the SEM run, participants had to perform a semantic categorization task. Specifically, they were required to judge whether the words (W) belonged to a living or a non-living category. In order to perform the language tasks, participants were instructed to internally pronounce the items, without any articulation or vocalization. During the control condition, participants had to perform a low level visual detection task. Specifically, they were asked to judge whether or not the unreadable words (UW) included at least one character that was of larger font size than the others. An example of each condition is illustrated in Figure 1. *Yes* and *no* responses were provided and transmitted by means of two manual keys pressed with the index and the middle finger of the dominant hand. Reaction time (RT) and response accuracy were recorded. Participants' responses were recorded and analyzed to ensure that participants performed the tasks correctly. Before the experiment, all participants went through a short training session outside the scanner with different items to those presented during the fMRI experiment.

### Functional MRI paradigm

A pseudo-randomized event-related fMRI paradigm, optimized (Friston et al., 1999) for 60 events (30 for each condition, i.e., language and control) and 35 additional null events, was used for the PHONO and the SEM runs. The two conditions of the PHONO run were: (a) PHONO (PW with and without the target phoneme) and (b) Control-PHONO (UW). The two conditions of the SEM run were: (a) SEM (W living and non-living) and (b) Control-SEM (UW). The null events were added in order to provide an appropriate baseline measure (Friston et al., 1999) and consisted of a white fixation cross displayed in the center of the black screen. A central fixation was also displayed between stimuli in order to encourage participants to fixate the center of the screen. The inter-stimulus interval was 2.5 s and the duration of each run was 8 min 40 s. Each run started with five initial dummy scans, in order to stabilize the magnetic field (corresponding images were discarded and not considered for the data processing).

### MRI acquisition

The experiment was performed in a whole-body 3T MR scanner (Bruker MedSpec S300) with 40 mT/m gradient strength at the Grenoble MRI facility IRMaGe in France. For functional runs the manufacturer-provided gradient-echo/T2^*^-weighted EPI method was used. Thirty-nine adjacent axial slices parallel to the bicommissural plane were acquired in interleaved mode. The slice thickness was 3.5 mm. During each session run the cerebral volume was measured 150 times. The in-plane voxel size was 3 × 3 mm (216 × 216 mm field of view acquired with a 72 × 72-pixel data matrix, reconstructed with zero filling to 128 × 128 pixels). The main sequence parameters were: TR = 2.5 s, TE = 40 ms, flip angle = 77°. To correct images for geometric distortions induced by local B0 inhomogeneity, a B0 field map was obtained from two gradient-echo data sets acquired with a standard 3D FLASH sequence (ΔTE = 9.1 ms). The field map was subsequently used during data processing. Finally, a T1-weighted high-resolution three-dimensional anatomical volume was acquired, by using a 3D-modified driven equilibrium Fourier transform (MDEFT) sequence (field of view: 256 × 224 × 176 mm; resolution: 1.333 × 1.750 × 1.375 mm; acquisition matrix: 192 × 128 × 128 pixels; reconstruction matrix: 256 × 128 × 128 pixels).

### Data analyses

#### Functional MRI processing

##### Spatial preprocessing

Data analysis was performed by using the general linear model (Friston et al., 1995) for event-related designs in SPM12 (Wellcome Department of Imaging Neuroscience, London, UK, www.fil.ion.ucl.ac.uk/spm) implemented in MATLAB (Mathworks Inc., Sherborn, MA, USA). Data analysis started by applying several spatial preprocessing steps. First, functional volumes were time-corrected with the 19th slice as reference (the brain volume was composed of 39 slices), in order to correct effects caused by the different acquisition time of each slice. Subsequently, all volumes were realigned to correct the head motion using rigid body transformations. The first volume of the first ER-fMRI session was taken as reference volume. T1-weighted anatomical volume was co-registered to mean images created by the realignment procedure and was normalized to the MNI space. The anatomical normalization parameters were subsequently used for the normalization of functional volumes. Finally, each functional volume was smoothed by an 8 mm FWHM (Full Width at Half Maximum) Gaussian kernel. Time series for each voxel were high-pass filtered (1/128 Hz cut-off) to remove low-frequency noise and signal drift.

##### Statistical analyses

The statistical analysis was subsequently performed on the spatially preprocessed data. For each participant we declared each task (PHONO and SEM) as a specific fMRI session and each session included two regressors (i.e., PHONO and Control-PHONO for the PHONO run; SEM and Control-SEM for the SEM run). Each of them was convolved with a canonical haemodynamic response function (HRF). Movement parameters derived from the realignment corrections (three translations and three rotations) were included in the design matrix as additional factors of no interest. The general linear model was then used to generate the parameter estimates of activity for each voxel, each condition and each participant. The statistical parametric maps were generated from linear contrasts between the HRF parameter estimates for the four experimental conditions. The spatial resolution of statistical parametric maps was the same as the spatial resolution of functional MR images (3 × 3 × 3.5). The statistical analyses were performed at individual and group levels. (A) Individual level: we computed contrasts enabling us to identify cerebral regions specifically involved in language processing after visual presentation of items. This allowed us to define regions of interest (ROI) that were subsequently included in a DCM analysis. For that purpose, we first calculated the main contrasts, Language vs. Control and Control vs. Language. The condition Language included both PHONO and SEM tasks. This contrast permitted the identification of cerebral regions specifically involved in language, including phonological and semantic processing. We also compared Language (PHONO and SEM) to Baseline. This was performed in order to identify visual areas involved in the tested language tasks and to include them as entry sites in our model. This particular contrast was chosen because the comparison Language vs. Control did not reveal any visual activation given that both conditions (Language and Control) were similar in terms of amount of visual information (see for instance Perrone-Bertolotti et al., 2014 for similar brain activity of high visual cortex during letters and unreadable characters). (B) Group level: contrast images obtained for individual analyses were entered into second-level random effect analyses to test for within-group effects (one-sample *t*-tests; Friston et al., 1998). According to the intensity of individual responses we retained clusters composed of at least 15 adjacent activated voxels for the Language vs. Control (*p* < 0.001 uncorrected, *T* = 3.48) and Language vs. Baseline (*p* < 0.05 FWE, *T* = 6.08) contrast. Clusters resulting from this random effect group analysis were used to define ROI for the DCM analysis (see Section ROI Construction and Time-Series Extraction). Form the Language vs. Baseline we only extracted activity within the vOTC region (MNI coordinates *x* = −39, *y* = −54, *z* = −14, according to Dehaene et al., 2010; Cattinelli et al., 2013; Perrone-Bertolotti et al., 2014)

#### Dynamic causal modeling analysis

DCM is a generic Bayesian framework used to infer directed connectivity between a predefined set of cortical regions (ROI), based on fMRI time series from these regions (Friston et al., 2003; Penny et al., 2004). More specifically, DCM enables estimation and inference of how one neural system (i.e., cerebral region) influences another within a defined network (i.e., model), and how it can be affected by the experimental context. Different parameters of these models are thus estimated at the neuronal level using a haemodynamic forward model, in order to compare the predicted functional responses (i.e., predicted with the defined models) to the measured responses (i.e., extracted time series). For a specified model, three sets of parameters are estimated: (i) driving input parameters that reflect how cortical regions respond to external stimuli (e.g., sensory stimulation), (ii) endogenous connection parameters that reflect the baseline effective connectivity between regions (i.e., what influence one region exerts over another), and (iii) modulatory parameters that quantify how effective connectivity is influenced by the experimental context (i.e., experimental conditions). These parameters are expressed in Hertz (Hz). For endogenous connections, they reflect how the rate of change in activity of one target region is influenced by the increase in activity in the source region. Positive (negative) values indicate that as the rate of change in activity in one region increases, activity in the target region increases (decreases). For modulatory connections, estimated parameter values are simply added to endogenous parameters and reflect context-induced change in connectivity.

##### ROI construction and time series extraction

**Figure 2:**
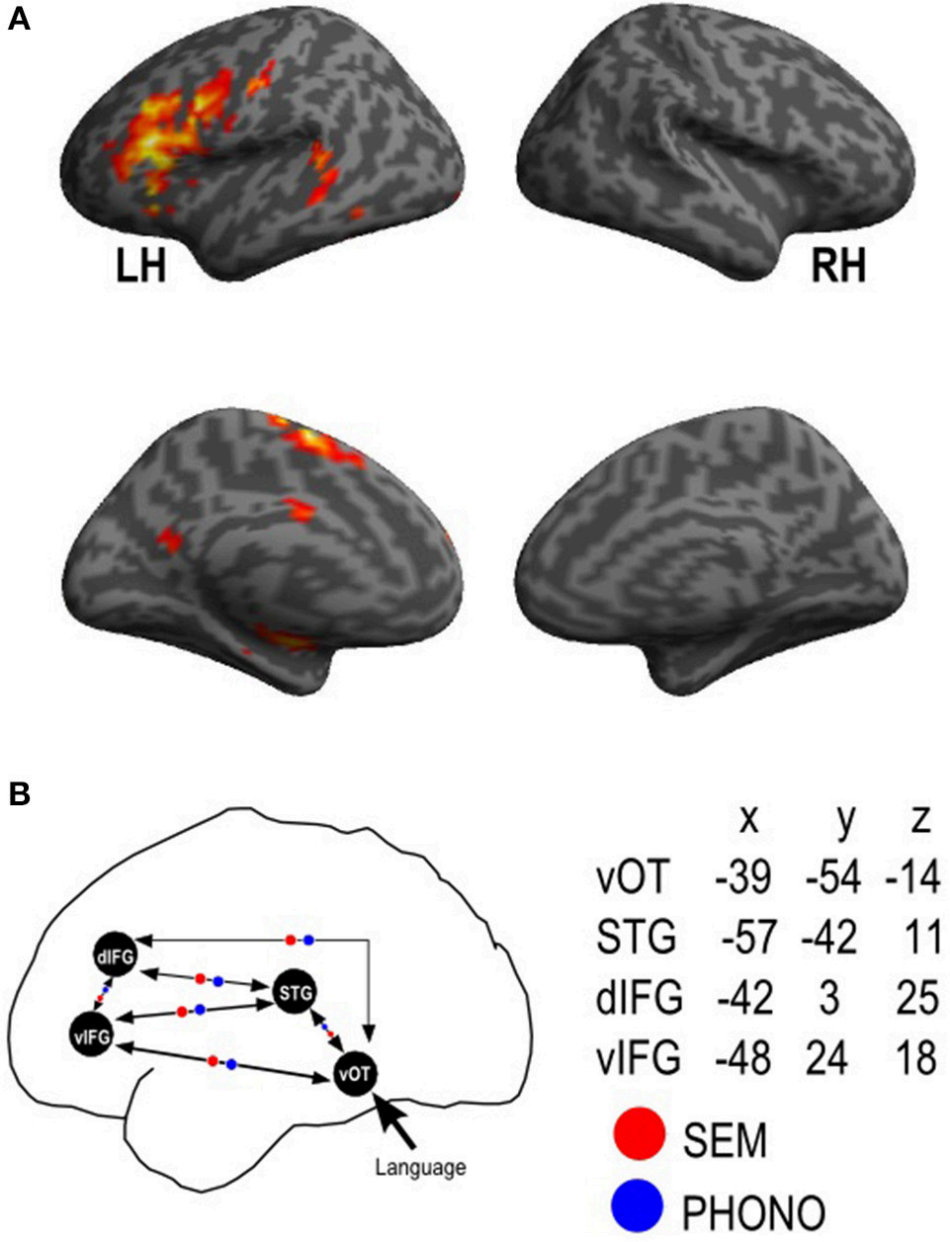
Functional MRI activation results and ROI dynamic causal model definition. fMRI results provided by the random-effect group analyses for the **(A)** Language vs. Control; Activation is projected onto lateral and medial surface rendering templates; **(B)** represents a schematic view of the connectivity model with the four regions of interest (vOT, STG, dIFG, vIFG, mean peak MNI coordinates were also presented) and reciprocal endogenous connections between all the regions. The external input (“Language”) reaches first the vOT region. Modulatory parameters are indicated in red for SEM (semantic) condition and in blue for PHONO (phonological) condition. LH, left hemisphere; RH, right hemisphere; vOT, ventral occipito-temporal cortex; STG, superior temporal gyrus; dIFG, dorsal inferior frontal gyrus; vIFG, ventral inferior frontal gyrus.

Consistently with our fMRI group analysis results and based on previous literature and research on PHONO and SEM processing (Perrone-Bertolotti et al., 2011; Vigneau et al., 2011; Price, 2012; Yvert et al., 2012), the Language vs. Control contrast allowed the identification of three ROI considered as hubs for our model. Furthermore, the contrast Language vs. Baseline provided a supplementary ROI, the visual cortex (i.e., which was defined as the input region of our model, see Construction of model section). Overall, with MarsBar software (http://marsbar.sourceforge.net/) we created in the left hemisphere four ROI's. To build them we defined spheres (8 mm radius) centered on the peak of activation of corresponding clusters provided by the group analysis. These four ROI taken into account were the vOTC, the STG, the dIFG, and the vIFG. These ROI and their MNI coordinates are illustrated in Figure 2. It should be noted that while the Language vs. Control contrast revealed a large cluster in the IFG encompassing its ventral and dorsal subdivisions, the dIFG and vIFG had distinct activation peaks within that cluster that were used to define these ROI.

Time series were concatenated across the two runs at the individual level. For each participant, and each region of interest, we first created a sphere (8 mm radius) centered on the peak of activation at the group level. We localized subject-specific maxima inside this sphere. We then extracted principle eigenvariates (adjusted for the participant's effect of interest) for voxels located in a smaller sphere (6-mm radius) centered on subject-specific maxima. Thus, for each ROI and each participant, we obtained a single time-series that included both the SEM and the PHONO runs.

##### Model construction

This DCM analysis aimed to specifically address whether SEM and PHONO differentially modulated connectivity between the vOTC, the STG, the dIFG, and the vIFG. Since we were mainly interested in investigating differences in SEM and PHONO in all connectivity parameters rather than assessing which particular connectivity was modulated by SEM and/or PHONO, we specified a single model (see Figure 2), including bidirectional endogenous connectivity between all regions in accordance with anatomical data (e.g., Catani and De Schotten, 2008; Friederici and Gierhan, 2013). The PHONO and SEM conditions were grouped into a single driving input (i.e., word recognition in the visual modality) that entered the system at the vOTC level. Finally, we specified a modulation of connectivity between all regions by SEM and PHONO. Once specified, this model was estimated for each participant in order to obtain connectivity parameters. It should be noted that we did not apply Bayesian model selection prior to parameter estimation in order to identify the optimal structure (i.e., endogenous connectivity) because (i) the purpose of the present study was to perform inference on connectivity parameters rather than on the model endogenous connectivity and (ii) it is possible that Bayesian model selection points toward a model in which an endogenous connection between two regions is absent, suggesting no influence of one region on the other under baseline conditions. However, it is still possible that this connectivity changes according to the experimental context which could result in “triggering” the effective connectivity between the two regions (i.e., modulation of connectivity). Thus, BMS to identify the optimal structure in terms of endogenous connectivity could have precluded the examination of PHONO- and SEM-modulation of some connections, in case these were not present in the endogenous connectivity of an optimal model. The specification of a fully connected and modulated model therefore allowed for all possible combinations of inter-regional connections to be estimated and tested for a difference in modulation by SEM and PHONO.

## Results

### Behavioral results

Behavioral responses recorded during the fMRI experiment showed that participants performed the task correctly for both language conditions: PHONO (*M* = 92%, SD = 19%) and SEM (*M* = 88%; SD = 24%) and for both control conditions (Control-PHONO—*M* = 91%, SD = 24%—Control-SEM—*M* = 91%, SD = 25%). The correct response level was significantly above chance (50%) for both PHONO [*t*_(23)_ = 22.83, *p* < 2.63E-17] and SEM [*t*_(23)_ = 15.88, *p* < 1.54E-13] conditions. Furthermore, no significant difference was observed between the two conditions [*t*_(23)_ = 1.11, *p* < 0.2] in terms of accuracy. In line with literature, participants were slower [*t*_(23)_ = 2.70, *p* < 0.01] to respond in the PHONO (*M* = 1110.05, SD = 915.73) than in the SEM (*M* = 984.17, SD = 328.06) condition.

### Functional MRI results

The results provided by the Language vs. Control contrast are summarized in Table 1 and illustrated in Figure 2A. Our results showed that an activated language network was located exclusively in the left hemisphere and included: (a) *frontal regions:* supplementary motor area (SMA, BA 6), precentral gyrus (BA 4); dorsal part (pars opercularis) of the inferior frontal gyrus (IFG, BA 44) and the ventral part (pars orbitalis) of the IFG (BA 45/46); (b) *temporal regions:* superior/middle temporal gyrus (STG, MTG, BA 22/21). Additionally, the right cerebellum was also involved as well as of the left Lentiform nucleus (Putamen).

**Table 1:** Activation peaks provided by the random-effect group analysis of Language vs. Control and Language vs. Baseline.

	**aal-Label**	**Side**	**BA**	***k***	***x***	***y***	***z***	***t***
**LANGUAGE > CONTROL**								
**Frontal lobe**								
Medial Frontal gyrus	Supp_Motor_Area	L	6	106	−6	−3	67	8.77
[Inferior frontal gyrus]	Frontal_Inf_Oper	L	44/6	401	−42	3	25	7.33
[Inferior frontal gyrus]	Frontal_Inf_Tri	L	45/46	401	−48	24	18	7.33
Precentral gyrus	Postcentral	L	4	58	−57	−12	49	5.69
**Temporal lobe**								
Superior temporal gyrus	Temporal_Mid	L	22/21	41	−57	−42	11	4.71
**Sub-lobar**								
Lentiform Nucleus	Putamen	L		117	−27	−9	−7	5.62
**Cerebellum**								
Cerebellum posterior lobe	Cerebelum_7b	R		94	24	−75	−46	7.85
**LANGUAGE > BASELINE**								
**Frontal lobe**								
Medial Frontal Gyrus	Supp_Motor_Area	L	6	485	−6	−3	67	13.40
	Frontal_Mid	R		20	33	−3	56	7.57
	Frontal_Inf_Oper	R	44/6	66	48	12	35	9.10
Insula	Insula	R	13	35	36	27	4	8.15
**Parietal lobe**								
Postcentral Gyrus	SupraMarginal	L	40	34	−57	−21	25	9.46
Inferior Parietal Lobule	Angular	R	39	147	36	−51	53	8.88
**Sub-lobar**								
Thalamus		L		858	−21	−33	4	12.21
		R		42	24	−30	4	11.26
**Occipito-temporal lobe**								
Lingual gyrus	Occipital_Inf_	L	17	4,047	−15	−93	−7	18.97
[Fusiform gyrus (vOTC)]	Fusiform	L	37		−39	−54	−14	15.18

*For each region we mentioned the cerebral lobe, gyrus, region's label (aal-Label, according to Tzourio-Mazoyer et al., 2002) and the corresponding Brodmann's area (BA). For each cluster we mentioned the MNI coordinates (x, y, z), number of voxels (k), hemisphere (right, R; left, L) and statistical value (t scores). The statistical threshold for individual voxels was set at p < 0.001 uncorrected for the Language vs. Control contrast and at p < 0.05 FWE corrected for the Language vs. Baseline contrast; the cluster extend threshold was 15 voxels. It should be noted that for the Language vs. Control contrast, both inferior frontal gyrus coordinates reported did not correspond to the main activation peaks of separate clusters but were identified as subpeaks within a large cluster ([within brackets]).Similarly, for the Language vs. Baseline contrast, the vOTC did not correspond to the activation peak and was identified within this large cluster ([within brackets]) according to literature results on the visual word form area*.

### DCM results

**Figure 3:**
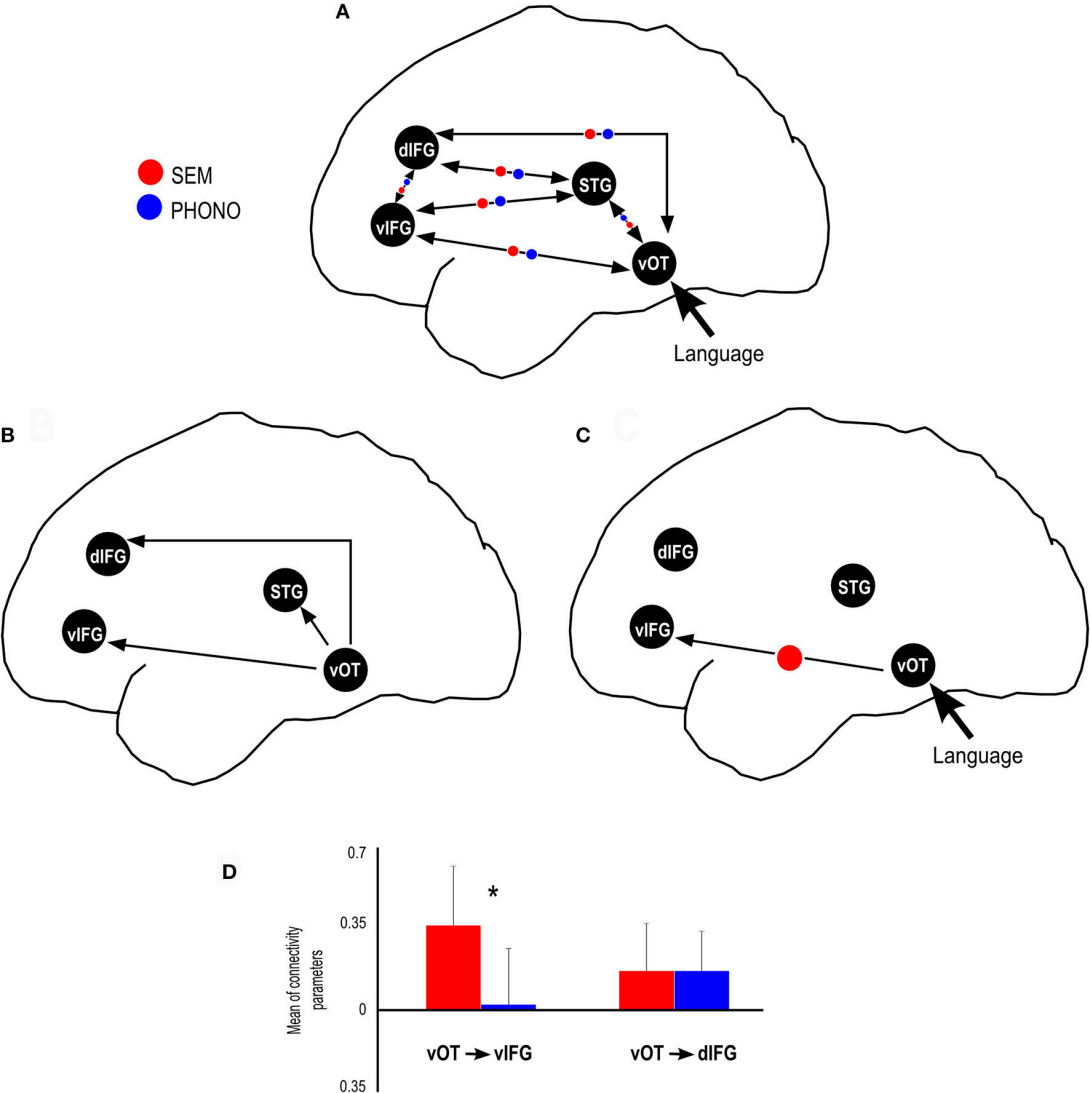
Summary of results provided by the dynamic causal modeling. **(A)** Schematic view of the connectivity model tested in this study, including the modulatory parameters for SEM and PHONO condition. **(B)** Significant endogenous connectivity parameters, showing that vOT activity results in an increase in STG, dIFG and vIFG activity. **(C)** Significant SEM (semantic) and PHONO (phonological) modulatory parameters connectivity, showing that the connectivity from the vOT to vIFG was greater than baseline connectivity in the SEM condition. **(D)** SEM condition modulated the connectivity from vOT to vIFG more strongly than PHONO condition and connectivity from vOT to dIFG was similar for SEM and PHONO conditions. ^*^Represents significant statistical difference.

Figure 3 is an illustration that synthesizes the main results obtained in this study: activated regions taken into account for the specification of the dynamic causal model for phonological and semantic processing.

After ensuring that assumptions for using parametric tests were met, we performed inference on connectivity parameters of our model using one-sample and paired *t*-tests, in order to assess (1) the significance of endogenous connectivity between all regions, regions (2) the significance of PHONO and SEM modulatory parameters and (3) differences in modulation of endogenous connectivity between SEM and PHONO. For endogenous connectivity, the significance of connectivity parameters was tested using one-sample *t*-tests against the null hypothesis that the connection strength equaled zero. Results for endogenous connectivity are summarized in Table 2 and Figure 3B. To assess the significance of PHONO and SEM modulatory parameters, we also used one-sample *t*-tests with the null hypothesis that each parameter is equal to 0. Finally, for modulatory parameters, we used paired *t*-tests in order to test significant differences in modulatory parameters between SEM and PHONO (results are summarized in Table 3 and Figure 3C). It should be noted that a paired *t*-test was the most appropriate here because each calculated connectivity parameter depends on every other parameter. Thus, it is a highly inter-dependent system of variables (i.e., connectivity parameters).

**Table 2:** Results for the endogenous connectivity parameters.

	**Mean**	**SD**	***t***	**df**	***p***	***d***
STG->vOTC	0.06	0.30	0.96	23	0.35	0.19
dIFG ->vOTC	−0.02	0.17	−0.56	23	0.58	−0.11
vIFG ->vOTC	−0.02	0.24	−0.42	23	0.68	−0.08
vOTC ->STG	**0.18**	**0.18**	**5.15**	23	**0.00**	**1.05**
dIFG ->STG	−0.06	0.15	−1.83	23	0.08	−0.37
vIFG ->STG	0.02	0.17	0.53	23	0.60	0.107
vOTC ->dIFG	**0.29**	**0.20**	**7.12**	23	**0.00**	**1.45**
STG ->dIFG	0.06	0.20	1.51	23	0.15	0.30
vIFG ->dIFG	−0.01	0.21	−0.29	23	0.77	−0.05
vOTC ->vIFG	**0.25**	**0.24**	**5.17**	23	**0.00**	**1.05**
STG ->vIFG	0.00	0.17	0.06	23	0.95	0.01
dIFG ->vIFG	0.03	0.22	0.56	23	0.58	0.11

*Table shows the descriptive statistical values (Mean and Standard deviation, SD) for each connectivity parameter and the statistical value for the one-sample t-test (including the t-value), the degree of freedom (df), the p-value and the Cohen's d effect size (d). Bold values represent significant statistical difference*.

**Table 3:** Results for the Semantic (SEM) and Phonological (PHONO) modulation parameters.

**SEM**		**One-sample** ***t*****-test**				**PHONO**		**One-sample** ***t-*****test**				**Paired** ***t-*****test**					
**Mean**	**SD**	***t***	**df**	***p***	***d***	**Mean**	**SD**	***t***	**df**	***p***	***d***	**Mean difference**	**SD difference**	**t**	**df**	***p***	***d***
0.03	1.09	0.11	22	0.90	0.02	0.18	1.68	0.52	22	0.60	0.10	−0.15	1.78	−0.43	23	0.67	−0.08
−0.50	1.51	−1.6	22	0.12	−0.32	−0.38	1.43	−1.29	22	0.20	−0.26	−0.12	2.37	−0.25	23	0.81	−0.05
−0.17	1.39	−0.59	22	0.55	−0.12	0.01	1.67	0.04	22	0.96	0.01	−0.19	2.24	−0.41	23	0.68	−0.08
0.20	1.02	0.96	22	0.34	0.19	0.33	1.05	1.53	22	0.13	0.31	−0.13	0.70	−0.90	23	0.38	−0.18
−0.37	1.13	−1.61	22	0.11	−0.32	−0.19	1.26	−0.72	22	0.47	−0.14	−0.19	1.06	−0.87	23	0.39	−0.17
−0.03	1.07	−0.11	22	0.90	−0.02	−0.17	1.34	−0.60	22	0.55	−0.12	0.14	1.40	0.50	23	0.62	0.10
0.16	1.14	0.7	22	0.48	0.14	0.16	0.81	0.95	22	0.35	0.19	0.01	0.64	0.06	23	0.96	0.01
−0.19	0.89	−1.024	22	0.31	−0.20	−0.05	1.45	−0.16	22	0.86	−0.03	−0.14	1.57	−0.43	23	0.67	−0.08
−0.06	1.17	−0.26	22	0.79	−0.05	−0.20	1.24	−0.79	22	0.43	−0.16	0.14	1.55	0.45	23	0.66	0.09
0.36	1.02	1.66	22	0.10	0.33	0.02	0.97	0.09	22	0.92	0.01	0.34	0.64	**2.55**	**23**	**0.02**	**0.52**
−0.03	1.40	−0.08	22	0.93	−0.01	−0.15	1.46	−0.51	22	0.61	−0.10	0.13	1.51	0.43	23	0.67	0.08
−0.37	1.05	−1.71	22	0.09	−0.34	−0.28	0.94	−1.47	22	0.15	−0.30	−0.08	1.12	−0.38	23	0.71	−0.07

*Table shows descriptive statistical values (Mean and Standard deviation, SD) for each SEM and PHONO modulation parameter and the statistical value for the one-sample t-test and paired t-test (including the t-value, the degree of freedom (df) and the p-value and the Cohen's d effect size (d). Bold values represent significant statistical difference*.

With regard to endogenous parameters (see Table 2), we observed significant positive connectivity from the vOTC to the STG [*M* = 0.18 Hz; *t*_(23)_ = 5.15, *p* < 0.05, *d* = 1.05], dIFG [*M* = 0.29 Hz; *t*_(23)_ = 7.12, *p* < 0.05, *d* = 1.45] and vIFG [*M* = 0.25 Hz; *t*_(23)_ = 5.17, *p* > 0.05, *d* = 1.05], indicating that an increase in vOTC activity results in an increase in STG, dIFG, and vIFG activity, irrespective of the experimental manipulations. These effects survived Bonferroni correction for the number of tested connections (*n* = 12, α/12 = 0.0042). All other endogenous connectivity parameters were not significantly different from 0, suggesting no effective baseline connectivity between the concerned regions. Regarding modulatory parameters, we observed that none of the modulatory parameters were significant different from the baseline for SEM and for PHONO. Nevertheless, the comparison of PHONO vs. SEM modulatory parameters for each connectivity link between regions revealed that SEM modulated connectivity from the vOTC to the vIFG significantly more than PHONO [SEM: 0.36 Hz; PHONO: 0.02 Hz; *t*_(23)_ = 2.55, *p* < 0.05, *d* = 0.52]. As modulatory parameters add up to endogenous parameters, this means that in the SEM condition, the connectivity from the vOTC to the vIFG was greater than in the PHONO condition. This effect did not survive Bonferroni correction. There was no other significant difference between PHONO and SEM modulatory parameters regarding connectivity between regions (all *ps* > 0.05), suggesting that PHONO and SEM did not differentially affect these connectivity measures between regions, including the vOTC to the dIFG (see Figure 3D). It should be noted that there have been some discrepancies in the DCM literature about whether or not one should correct inference on parameters for the number of tested connections. Indeed, although multiple comparisons are performed and would require correction for the number of tests performed, such correction might be too conservative because as mentioned previously, estimated parameters are highly dependent on each other (Stephan et al., 2010). Some authors even argued that a correction for multiple comparisons would be inaccurate (Parker-Jones et al., 2013; Kawabata-Duncan et al., 2014). As there is still no consensus on that issue, we believe that it is worth reporting both approaches, each having its potential limitations.

## Discussion

The aim of this fMRI study was to evaluate the modulation of the ventral and dorsal pathways by phonological and semantic processing during visual word recognition within a common cerebral network. Our main working hypothesis was based on the dual-route model of word recognition (Coltheart et al., 2001) and on previous findings on visual word recognition (e.g., Jobard et al., 2003; Ben-Shachar et al., 2007). Overall, these studies suggested that the dorsal pathway is specifically involved in grapho-phonological conversion during phonological tasks, whereas the ventral pathway is rather involved in lexico-semantic access during semantic tasks. In the present study, a phonological vs. semantic model has been considered, with a dorsal pathway including the vOTC, STG, and dIFG, and a ventral pathway including the vOTC and vIFG.

We performed a DCM analysis to evaluate the inter-regional interactions and the modulation of ventral and dorsal pathways through a phonological and a semantic task using visual stimuli. Endogenous parameters (i.e., baseline connectivity between regions, irrespective of the experimental context) results suggested that there are at least three pathways from vOTC to other language regions that could sub-serve functional information transfer through inter-regional interactions (see Figure 3B). Indeed, we found significant effective connectivity from vOTC to STG, vIFG, and dIFG. Given that vOTC is known to be involved in the orthographic processing of visual words (e.g., Cohen et al., 2000; Dehaene et al., 2005), it is possible that these routes subsequently enable visual word recognition. The evaluation of these inter-regional interactions (endogenous parameters) suggests that these three paths are involved in visual word recognition after the visual information input (see Figure 3B). Indeed, the endogenous parameters of these three paths were significantly different from zero for vOTC → dIFG, for vOTC→ STG and for vOTC → vIFG (see Table 2). Moreover, all of these three parameters were positive, suggesting that increased vOTC activity induces an increase in dIFG, STG, and vIFG activities, regardless of language demands (semantic, phonological). These results are in line with other studies suggesting that the vOTC is anatomically connected to left perisylvian areas involved in language processing, suggesting a direct connection between visual and language areas (Bouhali et al., 2014). These connections are related to the heterogeneous functionality of the vOTC (Vinckier et al., 2007; Bouhali et al., 2014; Perrone-Bertolotti et al., 2014) and also related to the proposal by Hickok and Poeppel (2007) that the vOTC performs the link between phonological and semantic information. Our results are also in line with those reported by Cattinelli et al. (2013) suggesting also three different networks during single word reading according to lexicality.

In terms of task-related modulation parameters, our results support a direct ventral pathway, which is more involved in semantic than in phonological tasks. Indeed, even though there was no significant modulation relative to the baseline connectivity by either SEM or PHONO, we showed dissociations between these two types of language processing, reflected by the increase of connectivity strength from vOTC to vIFG (ventral pathway) during semantic categorization as compared to the phonological task. Dissociation in the modulation of connectivity by the task demands was not observed between the vOTC and the dIFG (dorsal pathway). However, it should be noted as a limitation of the present results that one fully connected and modulated model is the most complex one, which can result in an over-fit of the data (Seghier et al., 2010). Furthermore, because the purpose of the present study was to perform inference on modulatory parameters and not on model structure, BMS was not used here. Therefore, we cannot ascertain that connectivity parameters were estimated within the best model structure: the estimated parameters reflect the most likely parameters given this particular model and dataset. It is thus possible that models with different architecture could result in different connectivity parameters. Therefore, the present results are restricted to the structure of the single model tested here.

The significant modulation of the vOTC → vIFG connection strength according to task demands specifically increased during the semantic condition as compared to the phonological condition. These results are in agreement with several anatomical connectivity studies (e.g., Friederici, 2011; Cummine et al., 2013; Menjot De Champfleur et al., 2013) suggesting that the anatomical ventral stream encompasses three anatomical fasciculi (see e.g., Duffau et al., 2009; Dick and Tremblay, 2012): the inferior longitudinal (ILF), the uncinated (UF), and the inferior fronto-occipital (IFOF) fasciculi. In support of the ventral pathway, and in relation with our results, several studies suggest that the IFOF is a direct pathway enabling rapid connectivity from vOTC → vIFG (Duffau et al., 2005; Dick and Tremblay, 2012; Sarubbo et al., 2013). Vandermosten et al. (2012) showed a significant correlation between fractional anisotropy (FA) values of the left IFOF and orthographic processing performances. Authors suggest that the left IFOF supports the ventral pathway of reading involved in direct word access (lexical acces). IFOF connects occipital (associative extrastriate cortex) temporal and frontal cortices (Martino et al., 2010), suggesting that a visual input (word or picture) is first processed at an occipito-temporal level, and then directly transferred to the frontal cortex. This is in line with recent results obtained by our team, using EEG recordings, suggesting fast activation of frontal regions during reading (e.g., Yvert et al., 2012; Perrone-Bertolotti et al., 2014) and could explain our results in terms of inter-regional connectivity from vOTC → vIFG. Furthermore, using intraoperative electrical stimulation, Duffau et al. showed that the stimulation of IFOF induces semantic paraphasia (Duffau et al., 2005; Duffau, 2008). The authors suggested that IFOF supports the ventral stream of word processing and plays a major role in semantic analysis (Duffau, 2008; Duffau et al., 2005). Contrary to our expectation we failed to show a top-down modulation on this pathway. Indeed, as previously suggested we expected a frontal to occipito-temporal or temporal connectivity modulation as proposed in literature (see Song et al., 2012; Yvert et al., 2012; Perrone-Bertolotti et al., 2014). Specifically, regarding a top-down influence from the frontal to the vOTC Price and Devlin (2011) proposed that during reading, an integrative process combining the bottom-up flow of visual inputs and top-down influences from high level language regions (such as Broca's Area, e.g., Yvert et al., 2012) is observed and this could be reflected by the involvement of a connectivity modulation from the frontal to the vOTC. Authors suggested that this is related to predictions performed by the systems in relation with task demands. It is possible that during our study this was not observed due to less task demands involved. Indeed, it is possible that more difficult tasks induce more predictions and so induce stronger top-down modulations. Recently, we showed that this predictive mechanism is more prominent when participants were invited to read recently learned items than when they were invited to read overlearned items (Perrone-Bertolotti et al., 2014). This could thus explain the absence of top-down modulation in the present data. Indeed; the present study involved the presentation of overlearned items. Therefore, task-demands were low, needing less prediction.

Finally, we failed to find a task-specific modulation (phonological and semantic) along the dorsal pathway. This result could be explained by the specificity of the phonological task used in our study: phoneme detection in pseudo-words used here could have determined higher recruitment of orthographic rather than grapho-phonological strategy (see Yvert et al., 2012). In line with this interpretation, Heim et al. (2008, 2005) failed to show modulation of dIFG activity by task demands (lexical, phonological). In a subsequent re-analyses of their data using DCM (Heim et al., 2009) the authors showed that ITG → dIFG connectivity was independent of the task while ITG → vIFG connectivity was modulated by lexical task demands. The former observation is thus in line with our results and could indicate that the dIFG supports non-specific processing or may be a more complex function in language processing. Developing on the earlier maturation time of the ventral pathway with inferior frontal cortex as compared to the later emerging connection with dorsal regions, Brauer et al. (2013) suggested that the dorsal stream might play a more complex role in language beyond the known functional segregation between phonology vs. semantics.

## Conclusion

Our results obtained with fMRI and the DCM approach showed specific inter-regional connectivity within the visual word recognition network. Our results suggest that only the ventral stream (vOTC → vIFG) was modulated by task demands. Specifically, the ventral stream was more modulated by lexico-semantic than phonological processing; the dorsal stream (vOTC → STG and vOTC → dIFG) failed to show such a differentiated modulation according to the task demands. Both our experimental task settings and developmental cerebral maturation issues (Brauer et al., 2013) could explain these findings. Overall, our results confirmed the known lexico-semantic analysis specificity of the ventral stream, failing to support such function for the dorsal stream.

## Author contributions

MP and MB designed research; MP, MB, and CP performed research; MP, MB, LK, CP, JV analyzed data; MP, MB, LK, CP, JV wrote the paper.

### Conflict of interest statement

The authors declare that the research was conducted in the absence of any commercial or financial relationships that could be construed as a potential conflict of interest.
